# Chromatin Dynamics and Genetic Variation Combine to Regulate Innate Immune Memory

**Published:** 2020-07-28

**Authors:** Jennifer Kim, Annie Vogel Ciernia

**Affiliations:** 1 Graduate Program in Neuroscience, Djavad Mowafaghian Centre for Brain Health, University of British Columbia, Vancouver, Canada;; 2 Department of Biochemistry and Molecular Biology, Djavad Mowafaghian Centre for Brain Health, University of British Columbia, Vancouver, Canada

**Keywords:** Autism spectrum disorder, Microglia, Alzheimer’s disease

## Abstract

Recent work by Ciernia et al. (2020) identified how genetic and epigenetic mechanisms interact to regulate innate immune memory in bone marrow derived macrophages. The authors examined the BTBR strain, a naturally occurring mouse model of Autism Spectrum Disorder (ASD) that captures the complex genetics, behavioral and immune dysregulation found in the human disorder. Immune cell cultures from the BTBR strain compared to the standard C57 showed hyper-responsive immune gene expression that was linked to altered chromatin accessibility at sites with genetic differences between the strains. Together, findings from this work demonstrated that multiple levels of gene regulation likely dictate the formation of innate immune memory and are likely disrupted in immune cells in ASD. Future work will be needed to extend these findings to immune gene regulation in the brain and how changes in immune function are related to abnormal behaviors in brain disorders.

## COMMENTARY

Autism spectrum disorder (ASD) currently affects 1 in 66 children in Canada, making it one of the most prevalent forms of childhood neurodevelopmental abnormalities. ASD is characterized by impairments in social communication and the presence of restricted interests and repetitive behaviors [1]. Symptoms are highly heterogeneous varying from mild to severe and are further complicated by the frequent occurrence of comorbidities [2]. The underlying etiology of ASD is equally as complex and involves both environmental and genetic risk factors [3–5]. While ASD is highly heritable, the genetic architecture is complex with hundreds of gene variants and copy number variants (CNV) associated with ASD [6]. Together, genetic analysis can currently only identify a potentially causative genetic abnormality in 20% of clinically diagnosed ASD cases [7]. However, these genetic studies have identified several categories of genes that have been implicated in ASD, including synaptic genes and regulators of chromatin structure and transcription [8]. A variety of epigenetic mechanisms appear disrupted in ASD including DNA methylation [9–11], post-translational modifications of histone tails [12], and non-coding RNAs [13]. Studies identifying differentially expressed genes in postmortem ASD brain samples have identified misregulation of both neuronal and immune genes [13–16], suggesting that epigenetic regulation of interactions between innate immune responses and neuronal activity play a critical role in the etiology of ASD.

Children with ASD often display a variety of immune related abnormalities [17] including altered brain, cerebral spinal fluid, and blood cytokine expression [18], as well as changes in both peripheral immune cell populations [19] and alterations in microglia morphology and density [20–24]. Together, this evidence suggests a disruption in the critical link between the developing immune system and fetal brain. There has been growing investment in understanding how microglia, the resident innate immune cells of the brain, are impacted in brain disorders like ASD. Microglia are uniquely long-lived cells that self-renew over the lifespan [25,26], suggesting that processes dictating early microglial development can have long-lasting impacts on cellular function and disease development. In addition to their role as the brain’s resident immune cells, microglia play critical roles in maintaining normal brain function through interactions with other brain cell types to regulate neuronal cell number [27], shape brain circuitry [28,29], and fine-tune neuronal connections [30–32] throughout life.

In reaction to an inflammatory event, microglia rapidly upregulate expression of pro-inflammatory genes and adopt a less-ramified morphology conducive for increased phagocytosis. After the resolution of the inflammatory event, microglia can remain “ primed ”, a permissive state in which subsequent immune challenges produce exacerbated inflammatory responses. Microglial priming has been observed to exacerbate pathology in mouse models of aging [33,34], Multiple Sclerosis [35], Parkinson’s Disease [36], stroke [37,38], and Alzheimer’s Disease [39–41]. In contrast to priming, repeated exposure to pathogens or other infectious agents can produce a subsequent repression of immune activation (tolerance), preventing the development of chronic inflammation or sepsis. While the mechanisms regulating innate immune memory in microglia are poorly understood [38,42,43], more easily accessible peripheral macrophage populations have served as model systems for identifying epigenetic regulators of innate immune memory.

Recent work from our lab describes how changes in chromatin accessibility contribute to altered innate immune memory in the bone marrow derived macrophage (BMDM) cultures from the BTBR T+Itpr3tf/J (BTBR) mouse strain, a common model for ASD. BTBR mice were derived from an inbred strain carrying the a^t^ (nonagouti; black and tan) and wild type T (brachyury) mutations that were crossed with mice with the tufted (Itpr3^tf^) allele. This mouse strain shows impairments in social behaviors, increased repetitive behaviors [44–48], and numerous genetic [49] and anatomical [50] alterations compared to the standard C57BL/6J mouse, making it a widely used model for ASD [51]. In addition, there is increasing evidence that BTBR mice also show increased baseline inflammation similar to that observed in children with ASD [51–54] and hence may serve as a more representative model of multiple genetic hits combined with environmentally driven inflammation. BTBR mice show increased levels of multiple cytokines in the brain (IL-1β, IL-18, IL-33, IL-6, and IL-12) [52] as well as increases in microglial numbers [55] and expression of microglial activation markers.

The complexity of the BTBR model allowed us to examine how differences in strain genetics and epigenetics potentially combine to impact immune gene regulation. We specifically examined how BMDM from each strain responded to repeated treatments with lipopolysaccharide (LPS), a component of the outer wall of gram-negative bacteria. Repeated low dose exposures to LPS has previously been shown to induce an endotoxin tolerance [56], in which repeated exposures result in blunted pro-inflammatory gene expression responses. This paradigm has been widely used to study immune gene regulation, and suppression of pro-inflammatory genes during the formation of tolerance requires epigenetic regulation [56,57]. Following repeated treatment with LPS to mimic repeated bacterial infections, we validated previously observed hyper-responses in immune gene expression in the BTBR compared to C57. Many of the genes that were tolerized (repressed) in expression in response to repeated LPS in C57, failed to fully attenuate in the BTBR. To begin to identify potential mechanisms underlying the differences in gene regulation between the strains, we profiled chromatin accessibility using ATAC-sequencing. Previous work examining nucleosome positioning at the *Il6* promoter region demonstrated that changes in chromatin accessibility were key for induction of immune tolerance of *Il6* expression [56]. Consequently, we sought to test the hypothesis that differences in chromatin accessibility between the strains would help explain differences in gene expression.

We identified differentially accessible regions (DARs) between strains and LPS treatments. The majority of LPS responsive regions were similar between strains, but there was a significant subset that showed differential levels of accessibility in response to LPS between strains. For example, several hundred regions showed either higher or lower accessibility at baseline between the strains. These same regions then showed altered responses to subsequent LPS treatment. For example, these inaccessible regions in the BTBR at baseline failed to open in response to LPS. These baseline DARs were enriched for markers of active enhancers, suggesting that they are regulatory regions with differential regulation between strains. Similarly, a subset of regions that changed chromatin accessibility in response to repeated LPS in the C57, failed to change in the BTBR. There were also regions that were uniquely responsive in the BTBR to LPS, together indicating both a failure in chromatin dynamics in some regions and abnormal chromatin dynamics in others. These regions were significantly enriched for strain-specific genetic variants, supporting the hypothesis that differences in the BTBR genome underlie at least some of the altered chromatin responses to LPS treatment. The strain-specific genetic variants were predicted to alter transcription factor binding of a few known regulatory factors, but the majority of motifs were similarly represented between the strains across LPS treatments. This suggests that changes in chromatin accessibility may contribute to altered gene expression through mechanisms beyond simple transcription factor accessibility, such as altered histone modifications or long-range chromatin interactions.

Histone modifications have been demonstrated to play important roles in the regulation of innate immune memory [56–58]. In a model of β-glucan induced immune priming, H3K4me1 (monomethylation of the 4^th^ lysine residue of histone 3), a hallmark of enhancer regions [59–61], has been shown to increase in parallel with *de novo* H3K27ac (acetylation at the 27^th^ lysine residue of histone 3) marks at distal regulatory regions in macrophages [58]. Interestingly, H3K4me1 marks remained elevated despite the return of H3K27ac to baseline at these regions [43,58]. Histone methylation relaxes local chromatin structure, allowing for steric accessibility to transcriptional machinery and increased gene expression. Thus, the persistent accumulation of H3K4me1 has been implicated as an epigenetic mechanism of establishing long-term innate immune memory [43,57,58]. In another study, treatment with LPS delayed the deposition of H3K27ac and H3K4me3 (trimethylation of the 4^th^ lysine residue of histone 3), which predominantly marks active promoter regions [59,61], in macrophages at promoters of pro-inflammatory genes, which attenuated responses to a secondary LPS challenge and contributed to immune tolerance [57,62]. However, how well these findings translate to microglia is uncertain. Transcriptomic and epigenomic (ATAC-seq and ChIP-seq) analysis of resident macrophage populations across numerous tissues, including brain microglia, revealed tissue-of-origin specific signatures [63–65], indicating that microglia have unique epigenetic profiles that differentiate them from other macrophages.

While the literature surrounding histone modifications regulating innate immune memory has thus far focused mainly on H3K27ac and H3K4me3, there are undoubtedly numerous additional histone modifications and other epigenetic mechanisms that act in concert to regulate gene transcription. For example, long-range chromatin interactions have recently been shown to play important roles in various central nervous system disorders [66,67] and in innate immune memory [68]. In a foundational study of brain cell-type specific promoter-enhancer interactions, Nott et al. (2019) found that microglial enhancers were significantly enriched for Alzheimer’s Disease (AD) risk variants identified in two landmark large-scale genome-wide association studies [69,70]. Proximity ligation-assisted ChIP-seq (PLACseq), a method which identifies long-range chromatin interactions between specific regions, revealed that AD-risk variants were linked to distal active promoters rather than simply the most proximal gene promoter, as had been previously assumed. Furthermore, enhancers harboring AD-risk variants were PLAC-linked to active promoters of both known AD genes from GWAS studies and an extended subset of novel genes [66]. In ASD, genome-wide profiling of enhancer marks (H3K27ac) of 257 postmortem ASD and matched control brain samples, showed increased acetylation for genes involved in synaptic function and neuronal excitability and decreased acetylation for genes involved in immune process related to microglia [12]. ASD has also been linked to polymorphisms in enhancer regions including the 5p14.1 locus, a region that exhibits enhancer activity that regulates expression of neurons in an autism-associated mouse model [71,72]. These findings highlight the importance of interpreting changes in histone modifications in the larger context of promoter-enhancer interactions in establishing innate immune memory and immune gene regulation abnormalities in ASD.

Repeated exposure to LPS has been shown to result in priming and tolerance of responses to subsequent immune challenges [38,43,57], thus serving as a valuable model for studying basic mechanisms of innate immune memory. Microglia also appears to show both priming and tolerance to repeated peripheral injections of LPS, leading to changes in cellular morphology, phagocytosis activity and gene expression [37,38]. Innate immune priming and tolerance in the brain also appear to rely on epigenetic regulation of microglia [38]; however, how well these mechanisms mirror those observed in peripheral macrophages or cultured immune cells is unclear. Together the findings of Ciernia et al. (2020) help lay the ground work for understanding how genetics and epigenetics combine to alter immune function in cultured macrophages. Future work will be needed to mechanistically test how epigenetic mechanisms coordinate gene expression in microglia and how these mechanisms may be perturbed in brain disorders such as ASD.

## Figures and Tables

**Figure 1:**
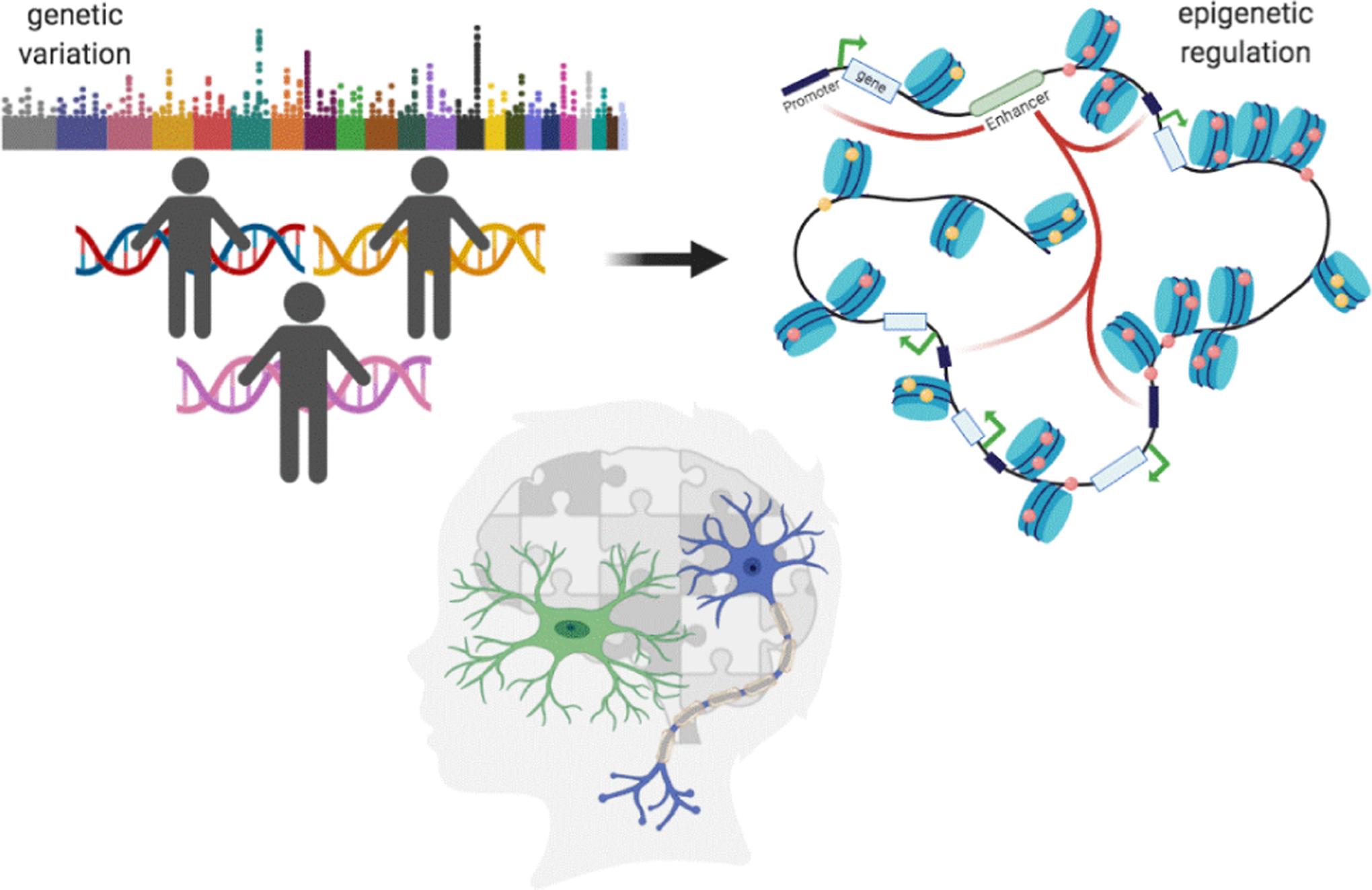
Genetic variation influences differential epigenetic regulation (histone modifications, long-range chromatin interactions) that shapes innate immune phenotypes in Autism Spectrum Disorder.
